# Curcumin prevents high-fat diet-induced hepatic steatosis in ApoE^−/−^ mice by improving intestinal barrier function and reducing endotoxin and liver TLR4/NF-κB inflammation

**DOI:** 10.1186/s12986-019-0410-3

**Published:** 2019-11-15

**Authors:** Dan Feng, Jun Zou, Dongfang Su, Haiyan Mai, Shanshan Zhang, Peiyang Li, Xiumei Zheng

**Affiliations:** 1 Guangdong Provincial Key Laboratory of Food, Nutrition and Health, Department of Preventive Medicine, School of Public Health, Sun Yat-sen University(Northern Campus), 74 Zhongshan Road 2, Guangzhou, 510080 Guangdong Province China; 2 Department of Cardiology, Affiliated Nanhai Hospital of Southern Medical University, Foshan, 528200 China; 3 Department of Clinic Nutrition, State Key Laboratory of Oncology in South China, Collaborative Innovation Center for Cancer Medicine, Sun Yat-sen University Cancer Center, Guangzhou, 510060 China; 4 Department of Clinic Nutrition, The First Affiliated Hospital, Sun Yat-sen University, Guangzhou, 510080 China

**Keywords:** Hepatic steatosis, Curcumin, Tight junction protein, Lipopolysaccharide, Toll like receptor 4, Nuclear factor-κB

## Abstract

**Background:**

Non-alcoholic fatty liver disease (NAFLD) is the most common chronic liver disease and has become a public health concern worldwide. The hallmark of NAFLD is hepatic steatosis. Therefore, there is an urgent need to develop new therapeutic strategies that are efficacious and have minimal side effects in hepatic steatosis and NAFLD treatment. The present study aimed to investigate the effect of dietary supplement of curcumin on high-fat diet (HFD)-induced hepatic steatosis and the underlying mechanism.

**Methods:**

ApoE^−/−^ mice were fed a normal diet, high-fat diet (HFD) or HFD supplemented with curcumin (0.1% w/w) for 16 weeks. Body and liver weight, blood biochemical.

parameters, and liver lipids were measured. Intestinal permeability, hepatic steatosis and mRNA and protein expressions of TLR4-related inflammatory signaling molecule were analyzed.

**Results:**

The administration of curcumin significantly prevented HFD-induced body weight gain and reduced liver weight. Curcumin attenuated hepatic steatosis along with improved serum lipid profile. Moreover, curcumin up-regulated the expression of intestinal tight junction protein zonula occluden-1 and occludin, which further improved gut barrier dysfunction and reduced circulating lipopolysaccharide levels. Curcumin also markedly down-regulated the protein expression of hepatic TLR4 and myeloid differentiation factor 88 (MyD88), inhibited p65 nuclear translocation and DNA binding activity of nuclear factor-κB (NF-κB) in the liver. In addition, the mRNA expression of hepatic tumour necrosis factor-α (TNF-α) and interleukin-1β (IL-1β) as well as the plasma levels of TNF-α and IL-1β were also lowered by curcumin treatment.

**Conclusion:**

These results indicated that curcumin protects against HFD-induced hepatic steatosis by improving intestinal barrier function and reducing endotoxin and liver TLR4/NF-κB inflammation. The ability of curcumin to inhibit hepatic steatosis portrayed its potential as effective dietry intervention for NAFLD prevention.

## Introduction

Non-alcoholic fatty liver disease (NAFLD) is the most common chronic liver disease, affecting 22–28% of the adult population and > 50% of obese individuals worldwide [1]. NAFLD covers a wide spectrum of liver pathologies which range from simple steatosis to non-alcoholic steatohepatitis. The hallmark of NAFLD is hepatic steatosis. Hepatic steatosis is a progression of excessive triglyceride accumulation caused by the imbalance between the influx and synthesis of hepatic lipids on one side and their β-oxidation and export on the other [2]. Many clinical and animal studies have indicated the central role of lipid accumulation in the progression and pathogenesis of NAFLD [3]. Therefore, it is an important step in the prevention of NAFLD to inhibit hepatic steatosis by reducing hepatic lipid accumulation.

The pathogenesis of hepatic steatosis is complex and has been shown to be associated with high-fat diet (HFD), obesity and a sedentary lifestyle, insulin resistance and type 2 diabetes [4]. Toll-like receptor-4 (TLR4) is a pattern recognition receptor of the innate immune system that plays a pivotal role in the innate immunity and inflammatory response [5]. Recent data has shown that TLR4 is also implicated in hepatic steatosis and NAFLD pathogenesis [6]. Loss-of-function TLR4 mutant mice are resistant to diet-induced NAFLD [7]. Ligands for TLR4 include gut-derived endotoxin lipopolysaccharide (LPS) [8], which is increased in different diet-induced rodent models of NAFLD [6]. LPS injections in NAFLD mice further increased proinflammatory cytokines and promoted liver injury [9]. High-fat diet can modify the gut permeability and elevate the serum LPS levels [10], and increased serum LPS can activate hepatic TLR4. Stimulation of TLR4 interacts with its downstream adaptor molecules myeloid differentiation factor 88 (MyD88) to activate nuclear factor-κB (NF-κB) transcription factor, subsequently results in production of proinflammatory cytokines such as tumor necrosis factor-α (TNF-α) and interleukin-1β (IL-1β), which propel the inflammatory reaction and cause the hepatic lipogenesis and lipid accumulation [6, 11]. Thus, strategies that reduce TLR4 ligand availability and/or inhibit hepatic TLR4 signaling would be expected to prevent hepatic steatosis.

Curcumin is a natural polyphenolic compound present in turmeric and possesses antiinflammatory, antioxidant and hepatoprotective properties [12, 13]. In recent animal studies, curcumin has been shown to have a protective effects on the liver against fat accumulation induced by a high-fat diet [14, 15]. The exact mechanism by which curcumin reduces liver fat accumulation and alleviates hepatic steatosis is not fully understood. TLR4 plays a pivotal role in hepatic fat accumulation and NAFLD development, several studies have shown that curcumin administration has been involved in the regulation of different inflammatory cytokines including TNF-α and IL-1β through inhibiting the activation of the TLR4/NF-κB signaling pathways [16, 17]. However, whether curcumin can prevents high-fat diet-induced fat accumulation and hepatic steatosis by inhibiting TLR4 signaling is still unknown. Therefore, the objective of this study was to investigate whether curcumin can attenuate HFD-induced hepatic steatosis and suppress NAFLD development in ApoE^−/−^ mice by improving intestinal barrier function and reducing TLR4 ligand availability and suppressing hepatic TLR4-mediated inflammation, as well as further investigate the protective effects of curcumin on atherosclerotic liver injury.

## Materials and methods

### Chemicals

Curcumin (purity≥98%) was obtained from Sigma-Aldrich (St. Louis, MO, USA). Antibody against TLR4 was purchased from Santa Cruz Biotechnology (Santa Cruz, CA). Antibodies against p65, occludin and β-actin were purchased from Cell Signaling Technology (Danvers, MA, USA). Antibodies against zonula occluden-1(ZO-1) and MyD88 were purchased from Abcam (Cambridge, MA, USA). SYBR Green-based real-time PCR kit was purchased from Applied Biosystems (Foster City, CA, USA). TRIzol reagent and the cDNA synthesis kit were obtained from Invitrogen Life Technology (Carlsbad, CA, USA). ELISA kits for TNF-α, IL-1β and LPS quantification were purchased from R&D Systems (Minneapolis, MN, USA). TransAM NF-κB p65 ELISA kit was purchased from Active Motif (Carlsbad, CA, USA).

### Animals and diets

Eight-week-old male ApoE^−/−^ mice with a C57/BL6 genetic background were obtained from the Beijing Vital River Laboratory Animal Technology Co. Ltd. (Beijing, People’s Republic of China). ApoE^−/−^ mice were fed in stainless steel metabolic cages at 22°Cwith a 12 h light/dark cycle with free access to food and water. After 1 week of adaptation, the mice were randomly divided into three groups (*n* = 10). The control group was fed a normal diet with 10% of energy as fat, the high-fat (HF) group was fed a high-fat diet (41% of total calories from fat; 0.15% cholesterol), and curcumin-treated group was fed a high-fat diet supplemented with curcumin (0.1% w/w). The dose of 0.1% curcumin was chosen according to previous study. Hasan et al. showed that curcumin at the medium dose of 0.05–0.1% was effective at reducing serum levels of several inflammatory cytokines [14, 15]. The compositions of the different diets were analysed chemically (Table 1). Following16 weeks of feeding, the ApoE^−/−^ mice were fasted overnight and then anesthetized. Blood samples were collected and the serum samples were separated by centrifuging the blood at 1500×g for 10 min at 4 °C. The entire liver and intestine were dissected out and weighed, some sections were snap frozen in liquid nitrogen prior to their storage at − 80 °C, and some sections were fixed in formalin or in cold acetone for further histological and immunohistochemistry analysis. All animal procedures conducted in this study were approved by the Animal Care and Use Committee of Sun Yat-sen University.

**Table 1:** Composition of the experiment mice diet

Ingredients (gm%)	Control	HF	HF + Curcumin
Casein	18.95	19.47	19.47
Corn starch	35.54	4.99	4.99
Maltodextrin	11.84	9.98	9.98
Sucrose	18.96	34.04	34.04
Cellulose	4.74	4.99	4.99
Oil	2.37	0.99	0.99
Fat	1.90	19.97	19.97
Mixed minerals, g	4.24	3.5	3.5
Mixed vitamins, g	0.95	1	1
Choline bitartrate, g	0	0.2	0.2
Cholesterol, g	0	0.15	0.15
Curcumin, g	0	0	0.1
Energy(kcal/g)	3.85	4.69	4.69
Energy from fat(%)	10%	41%	41%
Energy from carbohydrate (%)	70%	43%	43%
Energy from protein (%)	20%	17%	17%

The name of the diet and source, manufacturer: Diet MD 12016(10%fat); Diet MD12015 (41%fat); Medicience Ltd., China

Control, normal diet; HF, high-fat diet; HF + Curcumin, high-fat diet supplemented with curcumin

### Biochemical analysis

Total lipids were extracted from hepatic tissue according to the method of Bligh and Dyer. After evaporation to dryness under a stream of nitrogen, the lipid extracts were resuspended in a solution of 90% isopropanol and 10% Triton X-100. The total TG contents in the liver were then quantified using a commercial enzyme kits (BioSino, Beijing, China) on a Biosystem automatic biochemistry analyzer (Madrid, Spain) [18, 19]. Serum total cholesterol (TC) and TG were determined enzymatically by using commercial kits (BioSino,Beijing,China), according to the manufacturer’s recommendations. Serum alanine aminotransferase (ALT), aspartate aminotransferase (AST), low-density lipoprotein cholesterol (LDL-C) and high-density lipoprotein cholesterol (HDL-C) were determined with an automatic biochemistry analyzer (Olympus AU600, Tokyo, Japan) [18, 19].

### Histological examination

The liver slices were fixed with 10% buffered formalin, embedded in paraffin, cut at thickness of 5 μm and then stained with haematoxylin / eosin (H&E). For Oil Red O staining, livers were embedded in Tissue-Tek OCT, snap frozen and stored at − 80 °C. Images were captured using an Olympus BX60 camer at × 200 magnification. Steatosis was numerically scored following semi-quantitative pathological standard.

### Immunohistochemistry

To measure TLR4 expression in the hepatic tissues, immunohistochemical staining was used as previously described [20]. Briefly, liver sections were fixed in cold acetone for 10 min, washed with PBS for three times, and blocked with 3% BSA for 30 min at room temperature. After that, sections were incubated with specific antibodies (TLR4 antibody) overnight at 4 °C, then incubating with biotin-conjugated secondary antibodies, avidin-biotin complex, and DAB as a substrate. Finally, sections were counterstained with haematoxylin and then analysed with a Leica microscope (DM 2500, Leica, Bensheim, Germany).

### ELISA analysis

The plasma levels of TNF-α, IL-1β and LPS in the mice were assayed by the corresponding ELISA kits, following the manufacturer’s instructions. NF-κB activity was assayed as previously described [21]. In brief, nuclear extracts from mouse liver tissues were prepared, and the binding of p65 to DNA was measured with the TransAM NF-κB p65 ELISA kit, according to manufacturer’s instructions.

### Quantitative real-time PCR

Total RNA was extracted using Trizol reagents from mouse liver tissues. The levels of TNF-α and IL-1β mRNA were quantified by quantitative real-time PCR as previously described [21] . The primer sequences were shown in Table 2. The internal control used GAPDH mRNA.

**Table 2:** Primers used in real-time RT-PCR experiments

Primer Name	Sequence
TNF-α-F	5′- TGTAGCCCACGTCGTAGCAAA-3′
TNF-α-R	5′- GCTGGCACCACTAGTTGGTTGT-3’
IL-1β-F	5′-CAGCTTTCGACAGTGAGGAGA-3’
IL-1β-R	5′-TTGTCGAGATGCTGCTGTGA-3’
GAPDH-F	5′-ATGCTGGTGCCGAGTATGTTG-3’
GAPDH-R	5′-CAGAAGGTGCGGAGATGATGAC-3’

### Western blotting

The protein expression of TLR4, MyD88, ZO-1, occludin and nuclear p65 was detected by Western blotting. The methods for Western blotting was previously described [22]. Brieftly, proteins (50 μg) from mouse liver and ileal tissues or nuclear extracts were subjected to 7.5% SDS-PAGE and electrotransferred to a nitrocellulose membrane. The membrane was then immunoblotted with specific antibodies (TLR4, MyD88, ZO-1, occludin, p65) and secondary antibodies conjugated with horseradish peroxidase. The loading control used β-actin and LaminB antibody. The bands were visualized using Pierce™ ECL system, and the band density was determined by Image J software (NIH, USA).

### Statistical analysis

Data are presented as the mean ± standard error of the mean (SEM), all data were analysed with SPSS 25.0 for Windows (SPSS Inc., Chicago, IL, USA). Statistical analysis was performed using the unpaired Student’s t-test to test the mean of two groups, and one-way analysis of variance (ANOVA) followed by the Student-Newman-Keuls test was applied for comparisons between multiple experimental groups. A value of *P* < 0.05 was considered significant.

## Results

### Curcumin suppressed HFD-induced body and liver weight gain

The ApoE^−/−^ mice were fed a normal diet, high-fat diet and high-fat diet supplemented with curcumin for 16 weeks, the group with a normal diet was considered as control group. As shown in Table 3, the high-fat diet-fed mice, compared to controls, markedly gained weight, as well as significantly increased liver weight. Curcumin supplementation induced a significant reduction in body weight gain and liver weight, the body weight gain and liver weight of the curcumin group was significantly lower than that of the high fat (HF) group (*P* < 0.05). There was no difference in food intake among the three groups throughout the experimental period.

**Table 3:** Biochemical parameters for mice evaluated in this study

	Control	HF	HF + Curcumin
Body weight gain (g)	8.6 ± 2.5	15.1 ± 4.2 ^*a*^	9.8 ± 3.4^*b*^
Liver weight (g)	1.34 ± 0.09	1.96 ± 0.12 ^*a*^	1.53 ± 0.05^*b*^
Total food intake (g)	635.6 ± 22.3	637.9 ± 21.5	632.5 ± 20.8
ALT (U/L)	25.2 ± 3.8	30.5 ± 2.7 ^*a*^	28.7 ± 6.0
AST (U/L)	30.7 ± 5.4	65.4 ± 15 ^*a*^	42.8 ± 3.5 ^*b*^
TC (mmol/L)	22.54 ± 4.27	36.03 ± 3.43 ^*a*^	24.60 ± 6.64^*b*^
TG (mmol/L)	1.50 ± 0.80	1.96 ± 1.01 ^*a*^	1.69 ± 0.50
LDL-C (mmol/L)	18.76 ± 4.62	31.56 ± 4.81 ^*a*^	19.98 ± 5.24 ^*b*^
HDL-C (mmol/L)	7.29 ± 1.51	6.78 ± 1.46	9.07 ± 1.96 ^*b*^

Values are mean ± SEM (n = 10 per group). Control, normal diet; HF, high-fat diet; HF + Curcumin, high-fat diet supplemented with curcumin

^*a*^ Significant difference between the Control and HF groups(*p* < 0.05)

^*b*^Significant difference between the HF and HF + Curcumin groups (*p* < 0.05)

### Curcumin improved serum lipid profile in HFD-fed ApoE^−/−^ mice

As shown in Table 3, compared to control group, high-fat diet-fed mice showed significantly higher levels of serum TC, TG and LDL-C, and lower level of HDL-C. Curcumin treatment improved the high-fat diet-induced dyslipidemia, the levels of serum TC and LDL-C in curcumin group were remarkably lower than that in high fat group (*P* < 0.05), and the level of HDL-C in curcumin group was higher than that in high fat group (*P* < 0.05).

### Curcumin alleviated HFD-induced liver injury

To determine whether curcumin treatment could attenuate the high-fat diet-induced liver injury, the concentrations of serum ALT and AST were examined. As shown in Table 3,

the concentrations of serum ALT and AST in high-fat diet-fed mice were significantly higher than that in normal diet-fed mice. Curcumin administration significantly reduced the high-fat diet-induced elevation in serum AST(*P* < 0.05). Serum ALT in high fat group appeared to be higher than that in curcumin group, but there was no significant difference between the two groups.

### Curcumin attenuated HFD-induced hepatic steatosis

**Fig. 1:**
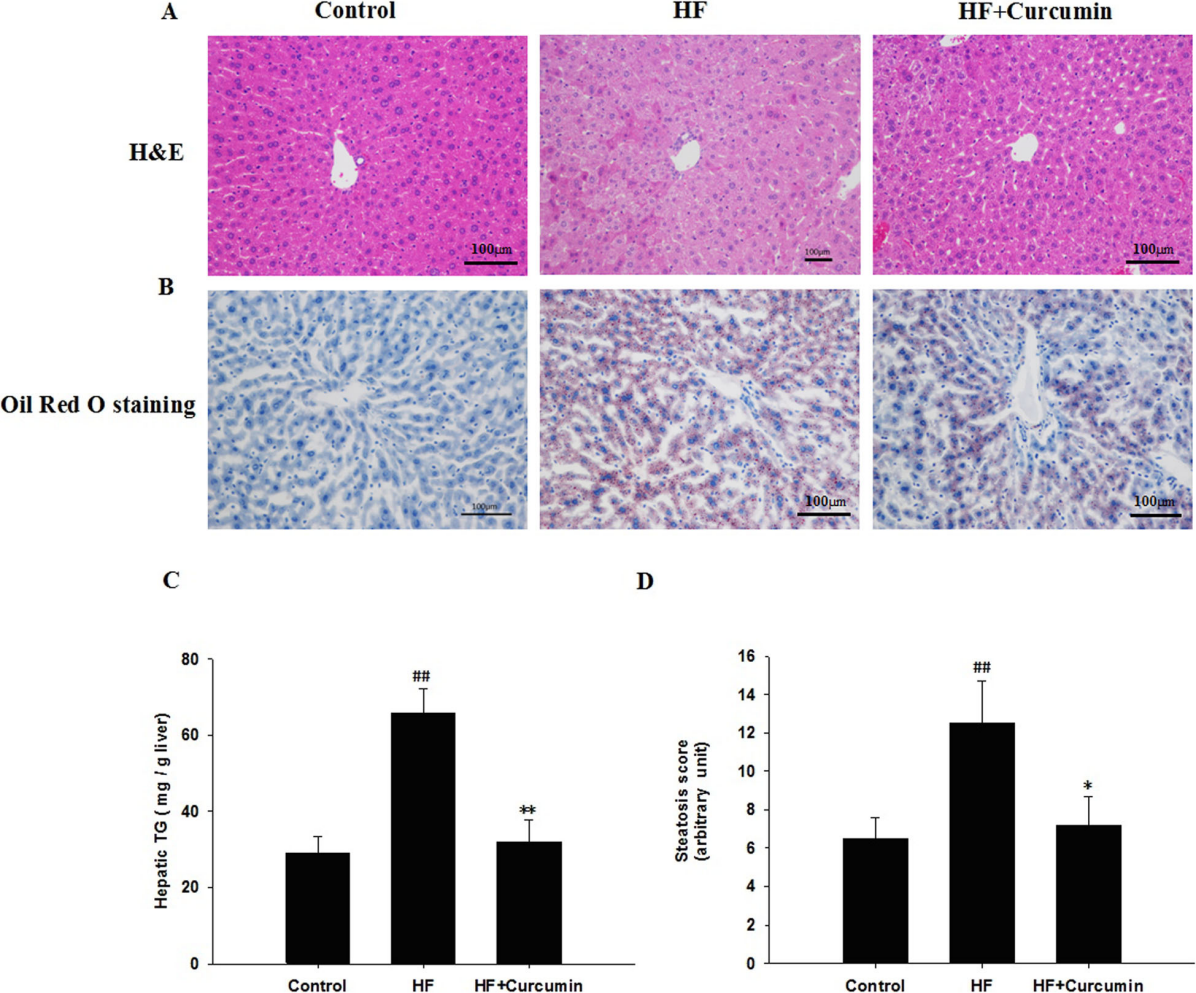
Effects of curcumin on liver histology and hepatic TG content in HFD-fed ApoE^−/−^ mice. ApoE^−/−^ mice were fed a normal diet, high-fat diet and high-fat diet supplemented with 0.1% curcumin (w/w) for 16 weeks, histological analysis of steatosis in liver sections stained with H&E (**a**) or Oil Red O (**b**) (magnification 200 ×). Hepatic TG content (**c**). Histological changes of steatosis in the liver were semi-quantitative and expressed as steatosis scores (**d**). Results are mean ± SEM (*n* = 10 per group). ^##^*P* < 0.01 versus control group; ^*^P < 0.05, ^**^P < 0.01 versus HF group. Control, normal diet; HF, high-fat diet; HF + Curcumin, high-fat diet supplemented with curcumin

The histological analyses by H&E (Fig. 1a) or Oil Red O staining (Fig. 1b) showed a remarkable increase of lipid deposition in the livers of high-fat diet-fed mice compared to those of normal diet-fed mice (Control group). Curcumin supplementation significantly reduced the high-fat diet-induced lipid deposition in the livers. Consistent with the results of histological analysis and liver weight, the hepatic TG concentration in curcumin-treated mice was about three fold lower than that in high-fat diet-fed mice (Fig. 1c). The degree of high-fat diet-induced hepatic steatosis was significantly attenuated in curcumin-treated mice, as shown by the decrease in the steatosis scores (Fig. 1d).

### Curcumin reduced serum LPS levels in HFD-fed ApoE^−/−^ mice

**Fig. 2:**
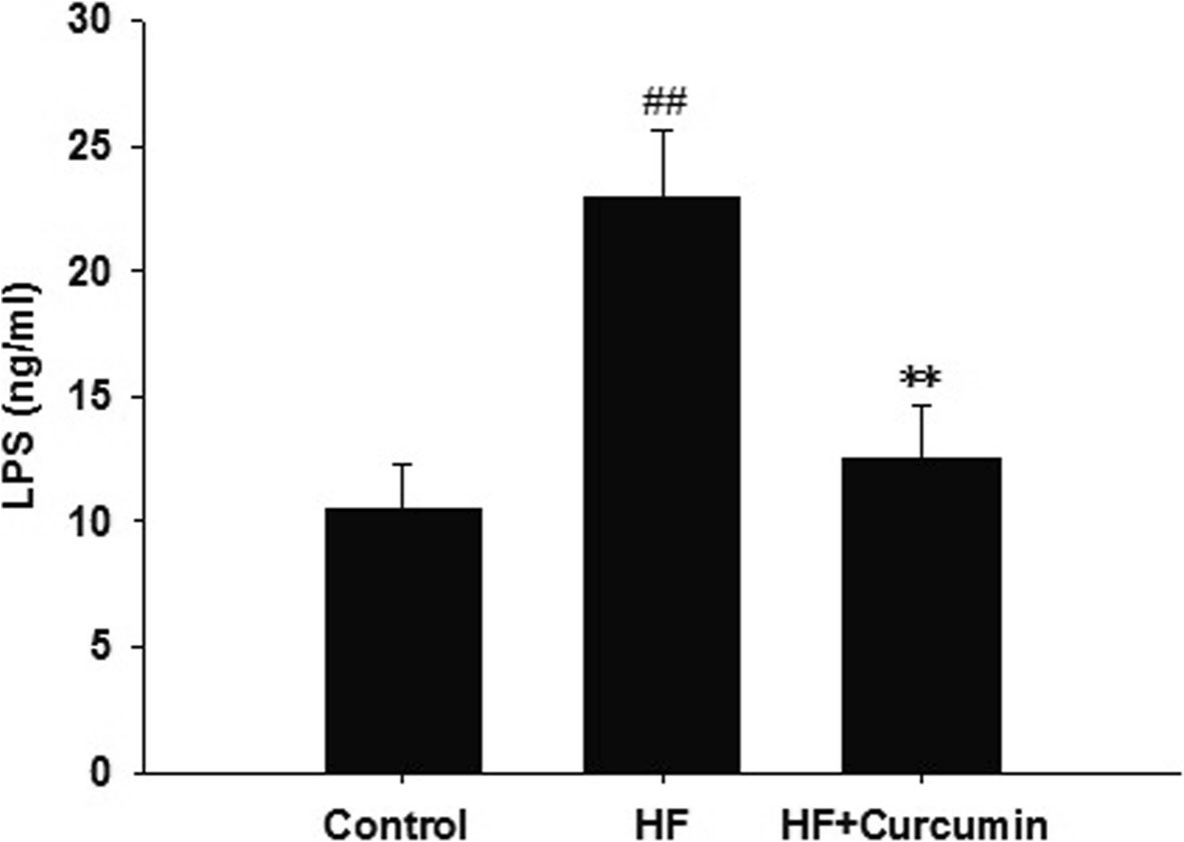
Effects of curcumin on circulating LPS levels in HFD-fed ApoE^−/−^ mice. ApoE^−/−^ mice were fed a normal diet, high-fat diet and high-fat diet supplemented with 0.1% curcumin (w/w) for 16 weeks, the serum LPS levels were measured by ELISA. Results are mean ± SEM (*n* = 10 per group). ^##^*P* < 0.01 versus control group; ^**^P < 0.01 versus HF group. Control, normal diet; HF, high-fat diet; HF + Curcumin, high-fat diet supplemented with curcumin

Endotoxin LPS derived from intestine functions as a natural ligand of TLR4 and is closely related with hepatic steatosis and the development of NAFLD [6], we thus examined that the impact of curcumin on circulating LPS levels. Compared to normal diet-fed mice, the serum levels of LPS were dramatically increased in high-fat diet-fed mice and reversed after curcumin administration (Fig. 2).

### Curcumin improved intestinal permeability in HFD-fed ApoE^−/−^ mice

**Fig. 3:**
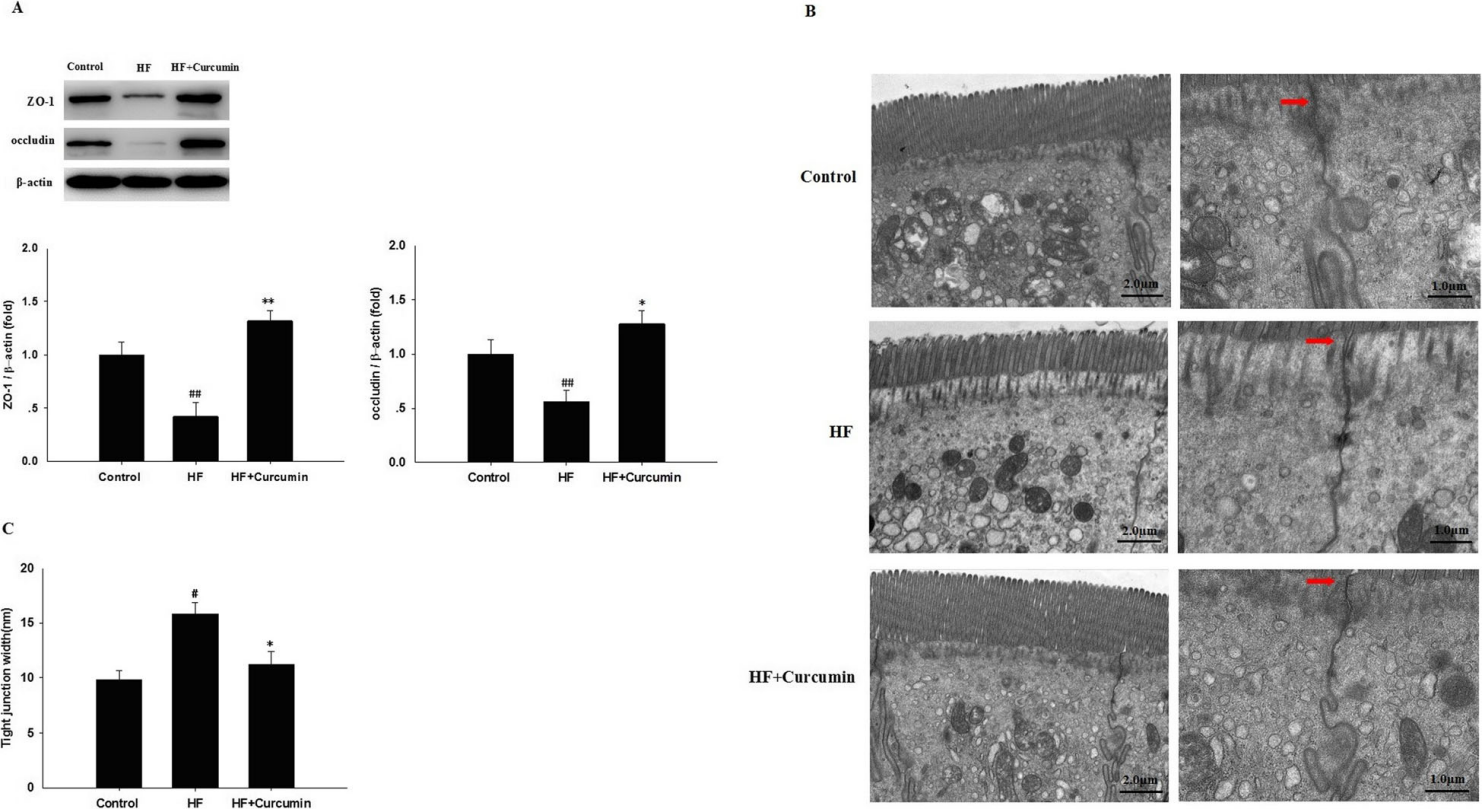
Effects of curcumin on intestinal permeability in HFD-fed ApoE^−/−^ mice. ApoE^−/−^ mice were fed a normal diet, high-fat diet and high-fat diet supplemented with 0.1% curcumin (w/w) for 16 weeks. (**a**) The protein expression of ZO-1 and occludin in ileal tissues was measured by Western blotting. (Top panel) Representative blot, (Bottom panel) Quantitative analysis of panel A. Results are mean ± SEM (*n* = 10 per group). ^##^*P* < 0.01 versus control group; ^*^P < 0.05, ^**^P < 0.01 versus HF group. (**b**) Ultrastructural observation of the tight junctions in the ileal mucosa and the width of the tight junction gap (transmission electron microscopy, 4000× or 8000×) (*n* = 10 per group). (**c**) The width of the tight junction gap (n = 10 per group). ^#^*P* < 0.05 versus control group; ^*^P < 0.05 versus HF group. Control, normal diet; HF, high-fat diet; HF + Curcumin, high-fat diet supplemented with curcumin

Since decreased expression of tight junction proteins, such as ZO-1 and occludin, leads to increased intestinal permeability and LPS translocation and plays an important role in the pathophysiology of NAFLD [23], we further determined the influence of curcumin on intestinal permeability. In comparision with normal diet-fed mice, the protein expression levels of ZO-1 and occludin in ileal tissues were markedly down-regulated in high-fat diet-fed mice, but restored following curcumin administration (Fig. 3a). To further evaluate the disruption of ileum microstructure, ileal tight junctions were examined by a transmission electron microscope (Fig. 3b). Compared with normal diet-fed mice, intact tight junctions in the ileal tissue were widened in high-fat diet-fed mice, but reversed by curcumin treatment (Fig. 3b and Fig. 3c). These results suggest that curcumin may improve intestinal barrier integrity in high-fat diet-fed mice.

### Curcumin reduced hepatic TLR4 and MyD88 expression in HFD-fed ApoE^−/−^ mice

**Fig. 4:**
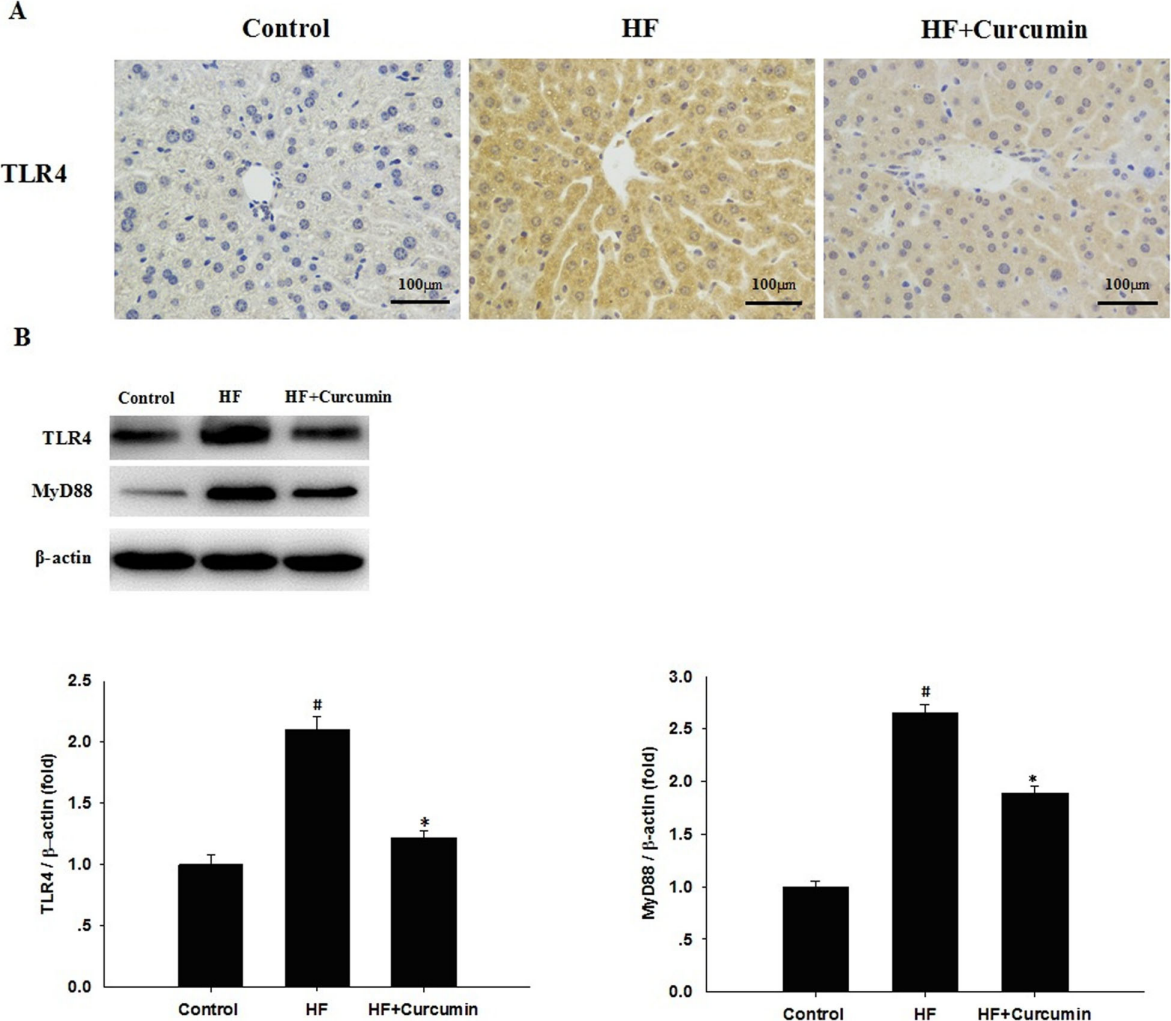
Effects of curcumin on hepatic TLR4 and MyD88 expression in HFD-fed ApoE^−/−^ mice. ApoE^−/−^ mice were fed a normal diet, high-fat diet and high-fat diet supplemented with 0.1% curcumin (w/w) for 16 weeks. (**a**) TLR4 expression in the liver was measured by immunohistochemical staining. Representative images of the control, HF and HF + curcumin groups (200 × magnification). (**b**) The protein expression levels of hepatic TLR4 and MyD88 were analyzed by Western blotting. (Top panel) Representative blot, (Bottom panel) Quantitative analysis of panel B. Results are mean ± SEM (*n* = 10 per group). ^#^*P* < 0.05 versus control group, ^*^P < 0.05 versus HF group. Control, normal diet; HF, high-fat diet; HF + Curcumin, high-fat diet supplemented with curcumin

Activating TLR4 signaling by LPS plays a critical role in the development of NAFLD [6]. To confirm the effects of curcumin on TLR4 signaling in the liver, immunohistochemical staining and Western blot with anti-TLR4 were performed to evaluate hepatic TLR4 and MyD88 expression. Compared to normal diet-fed mice, the immunohistochemical staining showed that the expression of TLR4 in the liver was markedly up-regulated in high-fat diet-fed mice, and restored after curcumin administration (Fig. 4a). Consistently, Western blot analysis further indicated that the upregulation of hepatic TLR4 expression induced by high-fat diet was reversed by curcumin treatment. In addition, curcumin supplementation significantly reduced the protein expression levels of hepatic MyD88 compared with high fat group (Fig. 4b).

### Curcumin suppressed hepatic NF-κB activation in HFD-fed ApoE^−/−^ mice

**Fig. 5:**
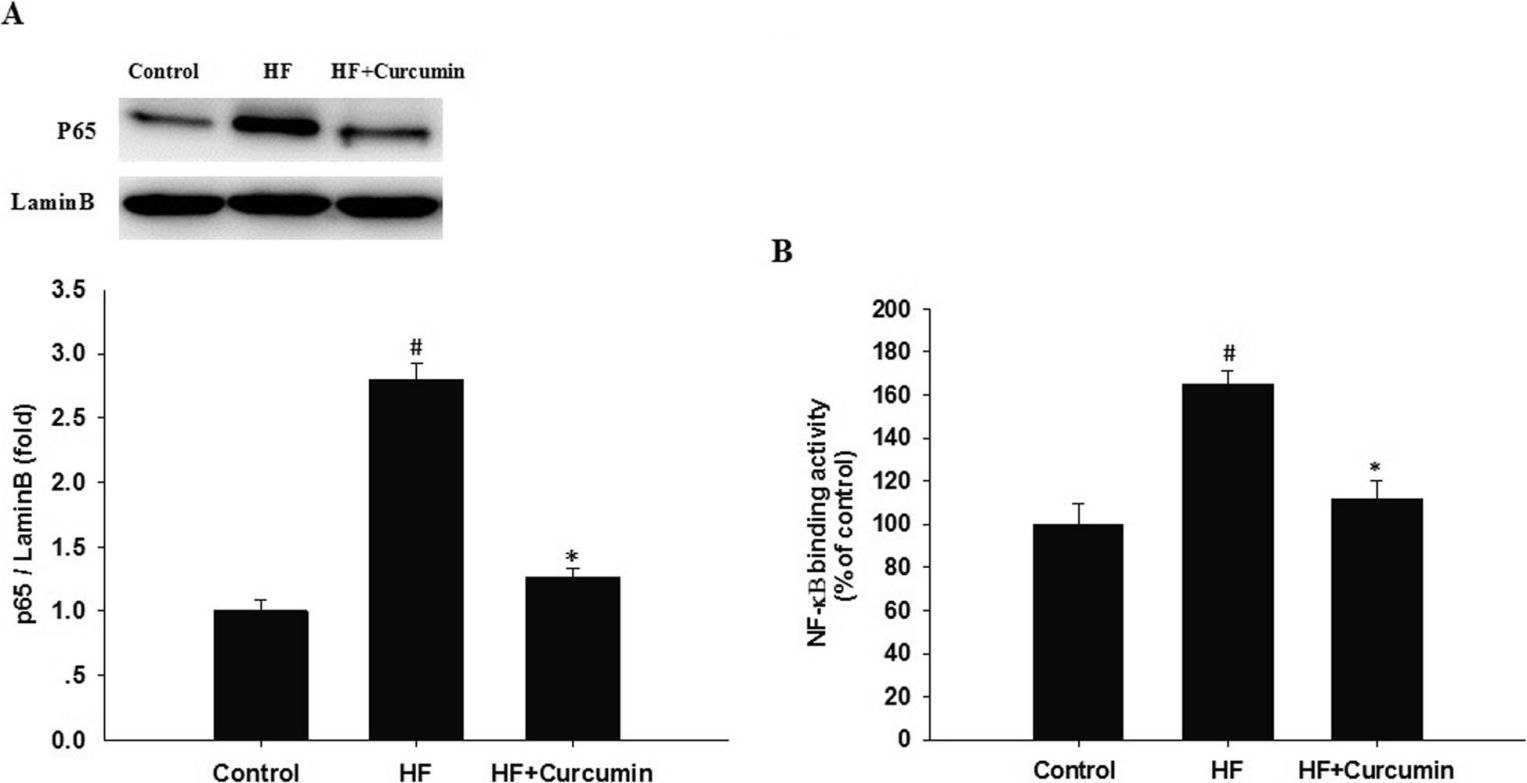
Effects of curcumin on hepatic NF-κB activation in HFD-fed ApoE^−/−^ mice. ApoE^−/−^ mice were fed a normal diet, high-fat diet and high-fat diet supplemented with 0.1% curcumin (w/w) for 16 weeks. Nuclear extracts from liver tissue were prepared for Western blotting of the p65 subunit of NF-κB (**a**) or NF-κB binding activity assay (**b**). For panel A, (Top panel) Representative blot, (Bottom panel) Quantitative analysis of panel A. Results are mean ± SEM (*n* = 10 per group). ^#^*P* < 0.05 versus control group, ^*^P < 0.05 versus HF group. Control, normal diet; HF, high-fat diet; HF + Curcumin, high-fat diet supplemented with curcumin

Stimulation of TLR4 results in the activation of NF-κB and subsequent transcription of proinflammatory genes [11]. To further insight into the anti-inflammatory effect and mechanism of curcumin, the nuclear translocation and DNA binding activity of NF-κB in the liver were examined. Compared to control group, nuclear proteins from the high fat group demonstrated significantly increased hepatic NF-κB p65 nuclear translocation, but restored following curcumin administration (Fig. 5a). Consistently, the increased DNA binding activity of NF-κB in high-fat diet-fed mice was also reduced by curcumin treatment (Fig. 5b).

### Curcumin reduced hepatic TNF-α and IL-1β expression in HFD-fed ApoE^−/−^ mice

**Fig. 6:**
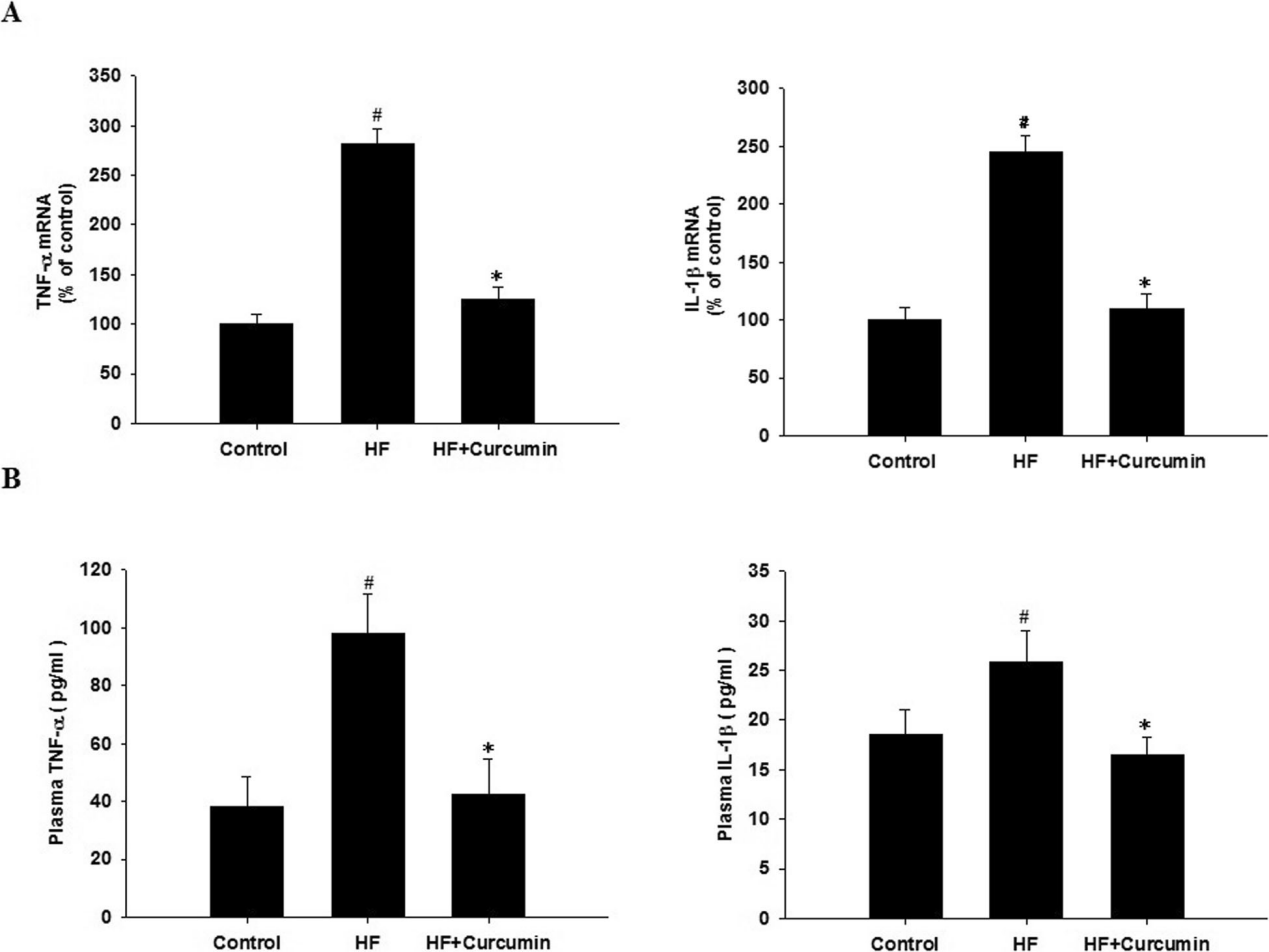
Effects of curcumin on hepatic TNF-α and IL-1β expression in HFD-fed ApoE^−/−^ mice. ApoE^−/−^ mice were fed a normal diet, high-fat diet and high-fat diet supplemented with 0.1% curcumin (w/w) for 16 weeks. (**a**) Hepatic TNF-α and IL-1β mRNA expression was analyzed by quantitative real-time PCR as described in the Materials and Methods. Expression values were normalized to housekeeping gene GAPDH. Results are mean ± SEM (n = 10 per group). ^#^*P* < 0.05 versus control group, ^*^P < 0.05 versus HF group. (**b**) The plasma TNF-α and IL-1β levels were measured by ELISA. Results are mean ± SEM (*n* = 10 per group). ^#^*P* < 0.05 versus control group, ^*^P < 0.05 versus HF group. Control, normal diet; HF, high-fat diet; HF + Curcumin, high-fat diet supplemented with curcumin

TNF-α and IL-1β have been shown to be the important proinflammatory cytokines involved in NAFLD development and can be released subsequently after activation of TLR4/NF-κB signaling pathway [6]. Therefore, we further determined the effects of curcumin on such cytokines. Compared to control group, the mRNA expression levels of TNF-α and IL-1β in the liver were markedly up-regulated in high fat group (Fig. 6a). Accordingly, the serum TNF-α and IL-1β levels were also increased by high-fat diet (Fig. 6b). Curcumin supplementation significantly reduced hepatic TNF-α and IL-1β expression and serum TNF-α and IL-1β levels induced by high-fat diet.

## Discussion

In the present study, we demonstrated that curcumin effectively prevents HFD-induced hepatic steatosis in ApoE^−/−^ mice. Moreover, our results suggest that curcumin treatment significantly inhibits HFD-induced hepatic fat accumulation by improving intestinal barrier function and reducing endotoxin and liver TLR4/NF-κB inflammation. To our best knowledge, this is the first in vivo study to reveal the molecular mechanisms of curcumin in preventing hepatic steatosis through modulating the gut-liver axis.

Non-alcoholic fatty liver disease covers a wide spectrum of liver pathologies which range from simple steatosis to non-alcoholic steatohepatitis. Hepatic steatosis is the hallmark of NAFLD and plays an essential role in the progression and pathogenesis of NAFLD [4]. Feeding animals with high-fat diet has been shown to induce obesity, metabolic syndrome and its hepatic manifestation, hepatic steatosis and NAFLD, mimicking the metabollicaly obese phenotype of Western countries [14, 15, 24]. In our study, HFD feeding induced body weight gain, dyslipidemia and liver lipid accumulation in mice. Hepatic steatosis was the main histopathological finding, as observed in other studies in mice fed a high-fat diet [15, 24, 25]. Oral supplementation with curcumin in HFD-fed mice counteracts increased liver weight by reducing liver steatosis derived from a diminished plasma dyslipidemia and hepatic triglyceride accumulation, suggesting the protective effect of curcumin on HFD-induced hepatic steatosis and NAFLD.

Although curcumin is known to exert positive effects on liver via multiple mechanisms, the precise mechanism responsible for its ability to alleviate liver steatosis remains incompletely defined. Previous research has already indicated that curcumin prevents HFD-induced dyslipidemia and steatosis by means of its modulatory effect on hepatic gene expression related to lipid metabolism, such as regulating AMPK activation and SREBP-mediated lipid biosynthesis [15, 26, 27]. Our current work demonstrated a novel mechanism of curcumin in preventing HFD-induced hepatic steatosis, i.e. to reduced gut-derived endotoxin translocation and hepatic TLR4/MyD88/NF-κB signaling pathway.

Endotoxin LPS derived from intestine functions as a natural ligand of TLR4, and altered TLR4 signaling is a key factor in the pathogenesis of NAFLD [6]. For instance, WT mice fed on a high-fat diet, fructose-rich diet, methionine/choline-deficient diet or choline-deficient amino acid-defined diet have shown steatosis/steatohepatitis with increased TLR4 expression and proinflammatory cytokines in the liver [7, 28–30]. Although the mechanism by which these diets induce steatosis is different, these diets modify the gut permeability and elevate the serum LPS levels [31, 32]. Additionally, continuously low-dose LPS injections in WT mice on standard laboratory chow resulted in hepatic weight gain and hepatic steatosis [30]. In contrast, loss-of-function TLR4 mutant mice are resistant to diet-induced NAFLD, even though LPS levels are equivalent to those in WT mice [7]. The plasma LPS levels are also elevated in NAFLD patients [33], and an high-fat diet elevates plasma LPS concentrations and its activity in humans [10, 34]. Thus, the LPS-TLR4 pathway plays a key role in the progression of NAFLD. In this study, we observed that curcumin administration significantly reduced the levels of circulating LPS. We then further examined the influence of curcumin on intestinal tight junction proteins expression such as occludin and zonula occluden-1, which are consistent with gut barrier dysfunction contributing to endotoxemia during NAFLD [23]. Curcumin treatment significantly upregulated ileal occludin and zonula occluden-1 expression along with lower levels of serum LPS and hepatic TLR4 expression. Our results indicated that curcumin prevented the translocation of gut-derived endotoxin LPS by reducing intestinal permeability and then lowered the ligand availability of TLR4, which also support that the anti-inflammatory and anti-NAFLD activities of curcumin occur along the gut-liver axis. In line with our results, several studies have reported that several polyphenols such as quercetin and green tea extract could prevent high-fat diet induced hepatic steatosis by modulating gut-liver axis [35, 36], gut-liver axis will be a potential target for NAFLD treatment.

Stimulation of TLR4 by LPS can interact with its downstream adaptor molecules MyD88 to activate NF-κB transcription factor and then induce the production of proinflammatory cytokines, which propel the inflammatory reaction and cause the hepatic lipogenesis and lipid accumulation. Our current study revealed that curcumin supplementation significantly down-regulated hepatic TLR4 and MyD88 expression, reduced p65 nuclear translocation and NF-κB DNA binding activity, indicating that curcumin suppressed HFD-induced the activation of TLR4-MyD88/ NF-κB signaling in the liver, and then prevented liver fat accumulation induced by high-fat diet in ApoE^−/−^ mice. Curcumin has been reported to regulate TLR4 signaling. For instance, Zhou et al. showed that curcumin modulated macrophage polarization by inhibiting TLR4-MAPK/NF-κB signaling pathway [37]. Wang et al. showed that curcumin suppressed LPS-induced sepsis in mice via inhibiting TLR4 signaling activation [38]. Curcumin was also found to exert an anti-inflammatory effect in rat vascular smooth muscle cells through suppressing ROS-related TLR4-MAPK/NF-κB signaling pathway [39], which were consistent with our results.

Activation of TLR4-MyD88/ NF-κB signaling results in the subsequent transcription of proinflammatory genes including TNF-α and IL-1β [11]. TNF-α and IL-1β are downstream targets of TLR4/NF-κB signaling and have been shown to promote the progression of NAFLD in animal models [40, 41]. In humans, the expressions of TNF-α and IL-1β as well as their receptor are increased in NAFLD patients [42, 43]. These data indicate that TNF-α and IL-1β are important mediators in the development of NAFLD. TNF-α has been shown to promotes triglycerides accumulation in hepatocytes, the mechanisms are considered to be related with impairing insulin signaling. Impairing insulin signaling results in insulin resistance with elevated insulin levels [44]. Insulin resistance increases serum levels of free fatty acid, and elevated insulin concentration facilitates free fatty acid flux into hepatocytes and hepatic lipogenesis [45]. Moreover, TNF-α promotes cholesterol accumulation in hepatocytes [46]. IL-1β is also involved in the progression of NAFLD including steatosis [41, 47]. IL-1β promotes hepatic triglycerides accumulation by suppressing PPARα and increasing the expression of diacylglycerol acyltransferase 2, an enzyme that converts diglycerides to triglycerides [47]. In the current study, the administration of curcumin significantly reduced the mRNA expression of hepatic TNF-α and IL-1β as well as the serum levels of TNF-α and IL-1β, which were accompanied by reduced inflammation and triglycerides accumulation in the liver. Therefore, the preventive effect of curcumin on hepatic steatosis is mediated, at least in part, by inhibiting hepatic TLR4-MyD88/NF-κB pathway and the subsequent production of TNF-α and IL-1β.

## Conclusion

In summary, we demonstrated in this study that dietary curcumin is an effective treatment for HFD-induced hepatic steatosis consistent with a mechanism of modulating the intestinal barrier function and related gut-liver axis activation. This work revealed a new mechanism related to gut-liver axis of curcumin in improving hepatic steatosis and suggested an important clinical application of curcumin in preventing NAFLD and atherosclerotic liver injury.

## Data Availability

Data sharing not applicable to this article as no datasets were generated.

## Abbreviations

ApoE^−/−^: apolipoprotein E-knockout
ALT: Alanine aminotransferase
AST: Aspartate aminotransferase
HFD: High-fat diet
HDL-C: High-density lipoprotein cholesterol
H&E: Haematoxylin / eosin
IL-1β: Interleukin-1β
LPS: Lipopolysaccharide
LDL-C: Low-density lipoprotein cholesterol
MyD88: Myeloid differentiation factor 88
NAFLD: Non-alcoholic fatty liver disease
NF-κB: Nuclear factor-κB
TLR4: Toll like receptor 4
TNF-α: Tumor necrosis factor-α
TG: Triglyceride
TC: Total cholesterol
WT: Wild type
ZO-1: Zonula occluden-1
